# Using Partially-Observed Facebook Networks to Develop a Peer-Based HIV Prevention Intervention: Case Study

**DOI:** 10.2196/11652

**Published:** 2018-09-14

**Authors:** Aditya Subhash Khanna, Steven Michael Goodreau, Stuart Michaels, John Alexis Schneider

**Affiliations:** 1 Chicago Center for HIV Elimination Department of Medicine The University of Chicago Chicago, IL United States; 2 Center for Studies in Demography and Ecology University of Washington Seattle, WA United States; 3 Department of Anthropology University of Washington Seattle, WA United States; 4 NORC The University of Chicago Chicago, IL United States

**Keywords:** African Americans, computer simulation, data mining, HIV infections, peer group, pre-exposure prophylaxis, sexual and gender minorities, social media, social networking

## Abstract

**Background:**

This is a case study from an HIV prevention project among young black men who have sex with men. Individual-level prevention interventions have had limited success among young black men who have sex with men, a population that is disproportionately affected by HIV; peer network–based interventions are a promising alternative. Facebook is an attractive digital platform because it enables broad characterization of social networks. There are, however, several challenges in using Facebook data for peer interventions, including the large size of Facebook networks, difficulty in assessing appropriate methods to identify candidate peer change agents, boundary specification issues, and partial observation of social network data.

**Objective:**

This study aimed to explore methodological challenges in using social Facebook networks to design peer network–based interventions for HIV prevention and present techniques to overcome these challenges.

**Methods:**

Our sample included 298 *uConnect* study respondents who answered a bio-behavioral survey in person and whose Facebook friend lists were downloaded (2013-2014). The study participants had over 180,000 total Facebook friends who were not involved in the study (*nonrespondents*). We did not observe friendships between these nonrespondents. Given the large number of nonrespondents whose networks were partially observed, a relational boundary was specified to select nonrespondents who were *well connected* to the study respondents and who may be more likely to influence the health behaviors of young black men who have sex with men. A stochastic model-based imputation technique, derived from the exponential random graph models, was applied to simulate 100 networks where unobserved friendships between nonrespondents were imputed. To identify peer change agents, the eigenvector centrality and keyplayer positive algorithms were used; both algorithms are suitable for identifying individuals in key network positions for information diffusion. For both algorithms, we assessed the sensitivity of identified peer change agents to the imputation model, the stability of identified peer change agents across the imputed networks, and the effect of the boundary specification on the identification of peer change agents.

**Results:**

All respondents and 78.9% (183/232) of nonrespondents selected as peer change agents by eigenvector on the imputed networks were also selected as peer change agents on the observed networks. For keyplayer, the agreement was much lower; 42.7% (47/110) and 35.3% (110/312) of respondent and nonrespondent peer change agents, respectively, selected on the imputed networks were also selected on the observed network. Eigenvector also produced a stable set of peer change agents across the 100 imputed networks and was much less sensitive to the specified relational boundary.

**Conclusions:**

Although we do not have a gold standard indicating which algorithm produces the most optimal set of peer change agents, the lower sensitivity of eigenvector centrality to key assumptions leads us to conclude that it may be preferable. The methods we employed to address the challenges in using Facebook networks may prove timely, given the rapidly increasing interest in using online social networks to improve population health.

## Introduction

### Background

Social network interventions have been successful in improving health outcomes [1-6], including those related to HIV prevention [7-9]. Our ongoing research aims to design social network interventions to reduce new HIV infections among young black men who have sex with men (YBMSM), defined here as individuals aged between 16 and 29 years. YBMSM are disproportionately impacted by the HIV epidemic in the United States [10], and traditional individual-level epidemiological interventions have had limited success in reducing HIV infections among YBMSM [11,12]. Peer-based interventions that make use of social networks have improved HIV outcomes in some populations [13-18] and present a promising opportunity to improve HIV outcomes among YBMSM. Here, we study how such a peer network–based intervention, which aims to expand the use of pre-exposure prophylaxis (PrEP)—a novel biomedical intervention with an estimated efficacy of over 90% among adherent individuals [19,20]—can be developed. PrEP remains underutilized among YBMSM, prompting a need to identify creative techniques to increase its use. Our objective here is to use online social network data from Facebook to identify influencers who could most effectively disseminate PrEP-related information among YBMSM in Chicago and to explore the methodological challenges that arise in the identification of these influential agents.

### Setting and Context

We use Facebook data from the *uConnect* cohort—the largest single-site population-based sample of YBMSM—to identify peer change agents (PCAs) who occupy *critical* positions in the social network. In future work, these PCAs will be invited to participate in training on how to effectively disseminate PrEP information. Although the use of alternate social networking sites has proliferated, Facebook remains an attractive choice because it is the most widely used social platform [21]. To identify PCAs, our population of interest is the potential influencers of YBMSM, who may or may not be YBMSM themselves, and Facebook enables broad characterization of their social networks.

We use a digital platform to characterize the social networks of YBMSM because despite demonstrating early promise, peer-based HIV interventions have had limited effect in some populations [22]. It has been argued that using digital methods to compile more accurate social network data and applying formal network analyses to identify PCAs may improve the efficacy of peer interventions [23]. As a digital platform, Facebook’s potential for improving health behaviors has been demonstrated in other studies [24-26], and Facebook continues to have high rates of use among sexual and gender minorities [27], including YBMSM in Chicago. (A more in-depth treatment of peer-based network interventions for HIV prevention is provided in Multimedia Appendix 1.)

There are a number of challenges, however, in using Facebook data to identify PCAs, including: (1) the large size of the Facebook network, which makes it problematic to identify which individuals are more likely to be influential among YBMSM; (2) difficulty in assessing the relative strengths of methods that can be used to identify PCAs; and (3) partial observation of the Facebook network that increases the uncertainty in identification of individuals in influential network positions. We address the aforementioned problems using a variety of techniques, including imputation to infer the unobserved structure of the Facebook network.

### Study Objectives

The goal of this paper is thus two-fold: (1) to provide insight into the structure of the Facebook network of YBMSM in Chicago and how that structure relates to the identification of PCAs for an HIV prevention intervention and (2) to provide guidance to researchers considering the use of PCAs on online social networks and the practical difficulties that might arise when applying theory to practice. We use 2 algorithms commonly used to identify candidate PCAs for information diffusion and apply them to observed and imputed networks. We examine the *sensitivity* and *stability* (defined in the Methods section below) of PCA sets selected by each algorithm, given the imputation of the unobserved data. This case study is the first step in a broader effort to understand how the effectiveness of peer-based network interventions can be improved. Methods and data presented here might be useful to other researchers using social networking sites for peer-based health interventions.

## Methods

### Recruitment of Study Sample

A detailed description of participant recruitment is provided elsewhere [28-31]. In brief, respondent-driven sampling (RDS) was used to recruit eligible YBMSM from the South Side of Chicago and adjacent suburbs between June 2013 and July 2014 (n=618) [28]. *Seeds* from diverse social spaces were selected and given coupons to distribute to potential recruits. If the recruits were willing to participate in the study, then they returned the coupons to the study coordinators and were given coupons to recruit study participants themselves. Information in these coupons allowed us to link the recruits with their recruiters. A diverse set of starting seeds for recruitment can produce a study sample that is representative of the population. RDS is a variant of link-tracing schemes, and it provides a design for sampling and a methodology for estimating statistical properties of the target population [32]. RDS is especially attractive for sampling populations that are *hard to reach*, and it has been used in a variety of health studies [33-39].

Respondents were eligible for recruitment if they (1) self-identified as African American or black; (2) were assigned male sex at birth; (3) were aged between 16 and 29 years; (4) spent most of their time on the South Side of Chicago or adjacent predominantly black suburbs; (5) were willing and able to provide informed consent at the time of the study visit; and (6) reported oral or anal sex with a male within the past 24 months [28]. These study participants answered a bio-behavioral survey at an in-person study visit and were offered the opportunity to provide Facebook data.

### Generation of Facebook Networks

Facebook friend lists of consenting uConnect participants were downloaded, allowing us to enumerate the set of potential influencers of YBMSM. An app within Facebook was developed to enable identification of unique individuals from Facebook friend lists of consenting respondents. With privacy protections in place, the algorithm unambiguously linked friend lists of all consenting uConnect respondents. Of the 618 study respondents, 600 reported using the internet and 490 reported having a profile on Facebook. Of the 322 who consented to provide Facebook data, 24 were not able to log in to their account. An undirected network dataset on 298 uConnect respondents was thus compiled, which included information on friendships between pairs of respondents and between respondents and *nonrespondents* (ie, friends of respondents who did not participate in the study). This data structure is typical of digitally collected network data [40,41].

**Figure 1:**
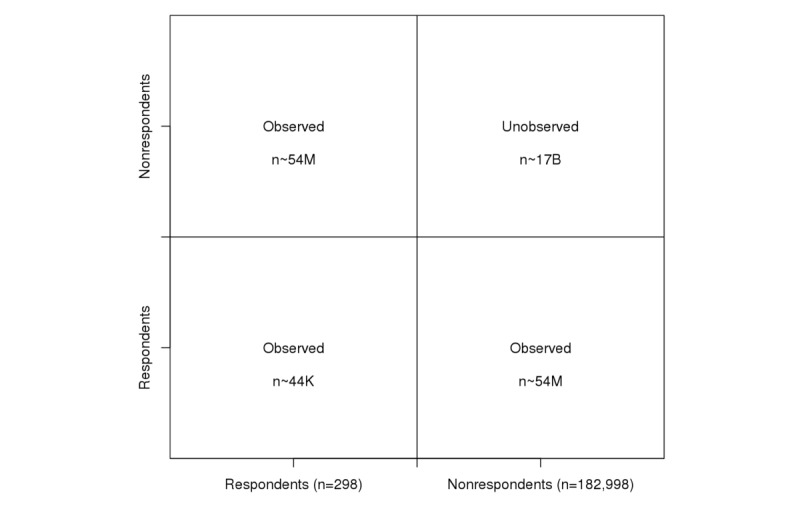
Illustration of the problem of unobserved tie imputation. Facebook friendships between individuals are classified into categories: observed respondent-respondent (bottom left quadrant), observed respondent-nonrespondent (diagonal quadrants), and unobserved nonrespondent-nonrespondent (top right quadrant). The approximate number of dyads in each quadrant is stated.

**Figure 2:**
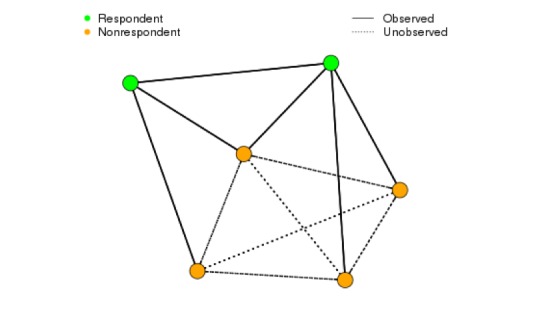
Illustration of the Facebook network data structure. Facebook friendships between respondents and between respondents and nonrespondents are observed. The friendships between nonrespondents are unobserved. Our data contain more nonrespondents than respondents.

Following Handcock and Gile [42], we depict the observed and unobserved partitions of our data in a 2x2 table (Figure 1). All friendships—*ties* or *edges* in network terminology—between respondents (n=298), as shown in the bottom left cell, were observed (about 44 thousand observed dyads), as were friendships between respondents and nonrespondents (n=182,998) in the diagonal cells (about 54 million observed dyads). Facebook friendships between the nonrespondent friends of uConnect respondents, shown in the top right cell, were unobserved (about 17 billion unobserved dyads). A schematic for the data structure is provided in Figure 2.

### Boundary Specification for Selection of Nonrespondent Nodes for Imputation

The Facebook network compiled above presumably includes nonrespondents that share variegated relationships with the respondents, including social, familial, and sexual, thus containing a mix of both strong and weak ties. Our goal is to identify critically positioned individuals, including those who were not respondents, and recruit them as candidate PCAs. For our intervention, it is not necessary that the nonrespondents be YBMSM themselves; it is only necessary that nonrespondents be potential influencers of YBMSM. Given the large number of nonrespondents and the amount of unobserved relational data between nonrespondents, we specified a boundary condition that would allow us to select individuals who were well connected to Chicago YBMSM.

We specified a boundary for nonrespondents that focused on the number of friendships with respondents (ie, their relations), as opposed to other individual-level criteria that are commonly used to specify boundaries [43,44], for the following 3 reasons. First, usage of the entire sample was infeasible, as there were some 17 billion unobserved dyads, and including all of these in our analyses would have meant that over 99% of our relational data were unobserved. Second, we had limited attribute data for the nonrespondents, and we assumed that those with large numbers of ties to YBMSM in South Chicago were the most likely to also be a potential influencer. Third, our ultimate goal was to select PCAs, and although degree is only one network criterion that determines the potential to influence other actors, it seems reasonable to expect most influential nodes will have moderately high degrees. Thus, we constructed a dataset that included all respondents and the set of nonrespondents who met the threshold specified by our relational boundary. Precise metrics on the relational boundary specification are given in the Results section below.

### Characterizing the Nature of Missingness in the Data

Although our Facebook networks were large, the information in our datasets was not complete because we did not observe the friendships between nonrespondents. The large amount of missing data could potentially bias our assessments of candidate PCAs based on their network position. Thus, we chose the approach of applying statistical imputation to reduce the bias induced by partial observation of our data.

To select an appropriate technique to impute the unobserved Facebook friendships, we first needed to define the nature of missingness in our data. We did this following the widely used convention developed by Rubin [45], who proposed that missingness is of 3 broad types. Data are “missing completely at random” (MCAR) when the missingness depends neither on the observed data nor the unobserved data. They are “missing at random” (MAR) when the probability of missingness does depend on the observed data but not the unobserved data [46,47]. Data are “missing not at random” (MNAR) if the probability of missingness depends on the unobserved data as well [46].

Our missing data are MNAR. Recall that our study respondents were recruited using an RDS procedure. Nonrespondents were Facebook friends of the respondents, and their data would not have been missing if they had been recruited into the study. However, nonrespondents might not have been recruited for the following unknowable reasons: (1) they were ineligible for the study; (2) they were eligible for the study, but the respondents did not wish to recruit them; (3) they were eligible for the study, but the respondents did not have sufficient coupons for them; and (4) nonrespondents received a coupon but did not participate in the study. Thus, the fact that friendship information for all pairs of nonrespondents is missing is related to their observed friendships with the respondents and also to their unobserved networks, as the nonrespondents’ reason for not being recruited cannot be determined. For reasons explained above, we also specified a degree-based boundary for nonrespondent inclusion. Thus, the respondents and nonrespondents have “differential popularity,” in the terminology of Gile and Handcock [48], beyond what can be explained by the observed data. Consequently, we suspect that the network structure of nonrespondents is different from that of the respondents. However, a difference in the network structure of respondents and nonrespondents by itself does not violate the MAR assumption, as long as the missingness is due to observed effects, such as whether a particular individual is a respondent or not. In our case, however, the difference in network structure is not entirely due to observed covariates; it may be attributable to a number of nodal covariates that were unobserved, as discussed above. Thus, the missingness in our data is consistent with the MNAR definition.

### Fitting a Model for Imputation of Unobserved Friendships

It has been shown that analyses using only the observed subnetwork might not generalize to the larger incomplete network [49]. Hence, an imputation of the unobserved Facebook friendships might provide more reliable indicators of candidate PCAs. To impute these unobserved friendships, we used exponential random graph models (ERGMs) [50], a class of models commonly used to describe probability distributions of networks, as implemented in the open source *statnet* [51] suite of packages. ERGMs were used to estimate the log-odds of ties between actor pairs, relative to a model where all ties are homogeneously distributed across the network. Observed existent ties were coded 1, observed nonexistent ties were coded 0, and unobserved ties were coded as missing (“NA” in *R*). This approach to imputation is derived from the method proposed by Handcock and Gile [42,48] and has been used to impute unobserved ties in other studies [52,53].

To impute friendships between nonrespondents, we developed a mechanistic model to predict their likelihood. Although there was little information available on individual attributes of nonrespondents (more details are provided in the Results), the number of friendships each of them shared with respondents was completely observed. We also know that Facebook ties tend to have a high mean degree and a high variance. It is, therefore, reasonable to assume that in the context of Facebook, nonrespondents who were more social with respondents might also be more social with each other. (A contrast would be the inverse condition, where observing many ties with respondents may indicate that many of the individual’s fixed *tie budget* was already used up, and thus, decrease their probability of having ties with other nonrespondents.) However, the procedure for selecting nonrespondents for imputation is biased toward those who are more social. It is, therefore, likely that the friendships between nonrespondents would not simply be in direct proportion to their observed friendships, but might experience a *dampening* effect. To mechanistically model both of these social forces, we used 2 separate parameters in an ERGM: *sociability* and *selective mixing*.

Sociability is a node-level parameter that measures the number of ties a respondent or nonrespondent shares with respondents, and selective mixing was represented as a single parameter measuring the number of ties between respondents and nonrespondents, as represented by either of the diagonal cells in Figure 1. The sociability term applies to the full adjacency matrix, allowing us to use the observed features to infer the unobserved [48]. It represents a process where respondents with more respondent friends will also have more nonrespondent friends. The selective mixing term allows us to model the systematic difference between the respondents and the nonrespondents, whereby the nonrespondent-nonrespondent quadrant will have a greater density than either of the other 2 (respondent-respondent and respondent-nonrespondent). This approach allows us to use the observed information to model the unobserved information in the network while accounting for the observed systematic differences between the respondents and nonrespondents directly, as advocated by Gile and Handcock [48]. Thus, the selective mixing parameter offsets some of the bias induced by selecting the most social nonrespondents as a consequence of the boundary specification defined above.

### Simulating Multiple Imputations From the Fitted Model

Once a model to impute missing data is estimated as above, we simulate stochastic realizations of this model. In networks that are fully observed, a simulation from an estimated ERGM fixes the set of nodes and uses a stochastic Markov Chain Monte Carlo (MCMC) algorithm to toggle relationships on and off, resulting in a network that represents a random draw from the probability distribution specified by the ERGM. In this case, we fix the values of the observed dyads, allowing only the unobserved dyads to be selected as candidates for toggling during the MCMC algorithm. This specification was used to impute 100 stochastically generated networks, each with the number of observed and unobserved ties consistent with a random draw from our fitted model described above. The 100 imputations were deemed to be sufficient because the maximum variability in the number of imputed edges was low (<1.8% of the mean, where mean=40,970 and range=40,610-41,340).

### Peer Change Agent Identification

Facebook friendships between nonrespondents are imputed in the simulated networks. We use these networks to identify PCAs. It is worth noting that the population that our PCAs are drawn from is not limited to YBMSM only but the potential influencers of YBMSM. Such a PCA identification procedure is arguably most successful when the type of flow process that is of interest is taken into account [54]. Following this argument, we apply 2 computational algorithms that are well suited to situations where the underlying flow process involves diffusion of information: eigenvector centrality [55] and keyplayer positive [56]. Eigenvector centrality assumes that the flow process of interest moves through the network via unrestricted walks. It describes a mechanism where 1 node can impact all of its neighbors simultaneously [54], and it has therefore been used in public health apps that use peer influence [57-59]. The keyplayer positive algorithm—henceforth referred to as *keyplayer* —is a set-based measure, reflecting the idea that the optimal set may not necessarily be composed of nodes that have the highest individual scores [56]. Rather, the keyplayer set consists of individuals who are maximally connected to individuals in the network. Thus, passing information through the keyplayer set minimizes the social distance it has to travel to reach the maximum number of individuals in a social network. Keyplayer is thus an ideal choice for scenarios like diffusing PrEP-related information, and it has been used in related public health applications [60,61]. Mathematical definitions and algorithmic descriptions of both measures are given in Multimedia Appendix 1.

We used eigenvector centrality and keyplayer to identify candidate PCAs on the observed Facebook data, in which all unobserved ties were assumed to be nonexistent, and then on each of the 100 imputed networks. These algorithms are designed to be applied to a given observed network, and the theory works best when the network observation is perfect. When networks are incompletely observed, however, an alternate approach is to apply each algorithm to a sample of imputed networks, rather than optimizing just on the observed dataset [56]. This method provides a set of PCAs that represent a good solution for the case where network data are imperfectly observed [56]. Thus, we followed this strategy.

We made a programmatic decision to select 300 individuals by each measure on the observed network, and the 300 most commonly occurring PCAs on the imputed networks were selected. The number of PCAs that are recruited and trained is a critical consideration in peer interventions, and it has been argued that a peer intervention is likely to be successful if the number of peer leaders recruited is about 7-8% of the size of the population for whom the intervention is designed [62]. In prior published work [63], we estimated that there are about 3700 HIV-negative YBMSM in Chicago, 8% of which is approximately 300.

We calculated the distribution of the number of times each individual was identified as a PCA across the imputations for each algorithm to assess which individuals warranted further consideration. These distributions were used to determine *cutoff* points for PCAs and were defined as a sufficiency condition for each of the algorithms. Of the identified PCAs, those that met this sufficiency condition were referred to as “sPCAs.” Intersection sets of PCAs on the observed network and sPCAs on the imputed network were then computed for each algorithm. We defined the following 2 measures to compare the sets of PCAs and sPCAs on the observed and imputed networks, respectively.

#### Sensitivity

The *sensitivity* of an algorithm is defined as the overlap in the PCAs identified on the observed network and sPCAs identified on the imputed networks. This measure allows us to assess if a PCA set differs substantially between the observed and imputed networks, providing an indication of the extent to which an individual appears to be a strong candidate for PCA selection, regardless of whether imputation is used. Thus, it helps us to understand the extent to which imputation affects our outcomes. Note that we do not assert that it provides an indication of the accuracy of the imputation, which remains unknowable.

#### Stability

The *stability* of an algorithm is defined as the tendency of an algorithm to identify the same nodes—that is, sPCAs on the imputed networks—across multiple imputations. This measure allows us to determine the threshold number of imputations for each measure that is required to select PCA sets of the size desired here (n=300).

### Note on Computing

All the computation and visualization were performed using software packages in the R programming language [64]. The *sna* [65] and *igraph* [66] packages were used to manage relational data. The *ergm* [67] package was used to fit the ERGMs and simulate the imputed networks. Eigenvector scores were computed using *igraph* [66]; keyplayer sets were identified using *influenceR* [68]. The *Intergraph* [69] package was used to convert data between the formats required for *igraph (or influenceR*, which uses the same data structure as *igraph)* and network (or *ergm*, which uses the same data structure as *network)*. The *sna* [65] and *GGally* [70] packages were used to visualize networks.

## Results

### Study Sample, Facebook Networks, and Relational Boundary Specification

The 298 uConnect respondents had 182,998 Facebook friends in total. There were 327,741 observed friendships in the dataset, including 3256 between respondents exclusively, and the remaining friendships existed between respondents and nonrespondents.

As stated above, as the number of nonrespondents was large and limited individual-level information on nonrespondents was available, we specified a boundary for nonrespondent inclusion based on their observed relations: nonrespondents (n=587) who were friends with at least 10.1% (30/298) of the respondents were included in our sample. The number of nonrespondents that would be selected with different boundaries is given in Table 1. Specifying a boundary involves tradeoffs; set too low, the amount of missing data increases rapidly (on the order of *n*^2^), and the likelihood of including someone not closely connected to Chicago YBMSM increases. By specifying a boundary that is too high, we may exclude someone who is both a YBMSM and a strong PCA candidate based on their network position. We considered the number of nonrespondents who would fall within different boundary specifications (Table 1) and the amount of missing data that each would imply. We selected 10% as our boundary for the number of respondents a nonrespondent must be friends with because it reduces our sample to a manageable computational size while retaining a broad sample of nonrespondents who could be PCAs. Thus, the final sample consisted of 885 individuals, including 33.6% (298/885) respondents and 66.3% (587/885) nonrespondents.

In this sample, the median reported age for both respondents and nonrespondents was 23 years (with 271 missing reports for nonrespondents). In addition, 96.9% (289/298) respondents and 92.5% (543/587) of nonrespondents identified their current gender as male on their Facebook profiles (2 nonrespondent reports were missing). Approximately 81.5% (243/298) respondents listed Chicago as their city on their Facebook profiles. Of the remaining 55 participants, 52 reported their residence as the South Side/south suburbs of Chicago during their in-person interview and 3 reported their residence as the Southeast side. Moreover, 66.2% (389/587) nonrespondents listed Chicago as their city; approximately 5.9% (35/587) nonrespondents did not report their city. About half of the remaining nonrespondents reported Illinois or another Midwestern State as their primary location, and the rest were scattered across the United States.

**Table 1:** Boundary specifications for the number of friendships between nonrespondents and respondents as a selection criterion for nonrespondents.

Minimum number of respondents that a nonrespondent has to be connected to (N=298), n (%)	Nonrespondents meeting that boundary specification, n	Observed friendships between respondents and nonrespondents, n	Unobserved dyads between nonrespondents, n
3 (1.0)	20,746	139,600	215,187,885
15 (5.0)	1633	47,473	1,332,528
30 (10.1)^a^	587^a^	26,444^a^	171,991^a^
60 (20.1)	97	5898	4656

^a^Shows the case used in our analysis.

**Table 2:** Summary of exponential random graph models fit.

Network parameter	Log odds	Standard error	*P* value
Edges	−5.36	0.029	<.001
Sociability (measured as degree with respondents)	0.044	0.0002	<.001
Mixing between respondents and nonrespondents	0.208	0.022	<.001

Overall, we observed 29,700 friendships, including 3256 between respondents exclusively and 26,444 between respondents and nonrespondents. Thus, each respondent had an average of 110.5 friendships, including 21.8 friendships on average with other respondents, and an average of 88.7 friendships with nonrespondents. Each nonrespondent who met our boundary specification had an average of 45.1 observed friendships.

The density of friendships—defined as the ratio of the number of observed friendships to the maximum number of possible friendships—between respondents was 7.4%. The density of respondent-nonrespondent friendships was 15.1%. (These densities will help us interpret outputs from our imputation procedure below.)

### Fitted Model for Imputation of Unobserved Friendships

Estimates from the fitted ERGM are in Table 2. The “edges” term operates analogously to an intercept term in logistic regression models. Our coefficient for it was negative, implying the base probability of a tie, without considering other additive terms, is less than 50%. Coefficients for the other 2 terms were positive and significant. The positive sociability coefficient indicates that individual respondents who have more ties to other respondents also have more ties to nonrespondents. The positive mixing coefficient indicates the dampening effect discussed above. Thus, the number of imputed friendships between nonrespondents was lower than that predicted by a proportional scaling model of the observed friendships between respondents and nonrespondents.

### Multiple Imputation of Unobserved Network Data

**Figure 3:**
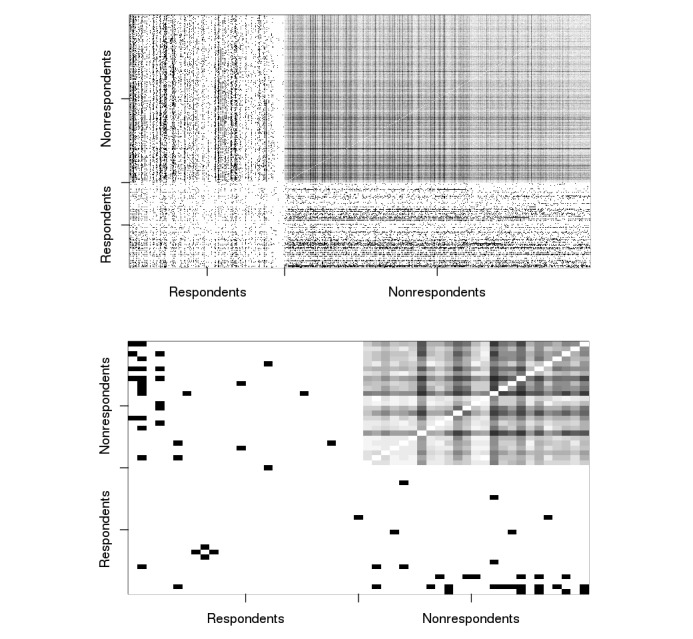
Frequency plots for imputed networks: the top figure displays data for all 885 nodes, and the bottom figure displays a subset comprising the first 50 respondents and first 50 nonrespondents (bottom), uConnect 2013-2014. The bottom left and the diagonals cells in both matrices consist entirely of observed dyads that required no imputation. The top right cell contains unobserved dyads, and edges in these dyads were stochastically imputed, and hence, appear in grey. The bottom panel is shown to produce a clearer display of the cell shading.

In Figure 3, the top panel contains frequency plots of the adjacency matrices across the 100 imputed networks. (The top panel of Figure 3 displays data for all 885 nodes; in the bottom panel, we selected the first 50 respondents and 50 nonrespondents to produce a clearer display.)

The mean density for imputed friendships between nonrespondents, shown in the top right corner of Figure 3, was 23.8%. The nonrespondent-nonrespondent density is higher than the density of respondent-respondent friendships (7.4%, as stated above) and the density of respondent-nonrespondent friendships (15.1%). This discrepancy in densities is reflective of the fact that a degree-based criterion was used to select nonrespondents for imputation. Had we not included the selective mixing term, however, this density would have been even higher. Three cells—the bottom left and the diagonals—in each panel of Figure 3 consist entirely of observed dyads and required no imputation. The top right cell contains unobserved dyads, and edges in these dyads were stochastically generated in the imputations.

**Figure 4:**
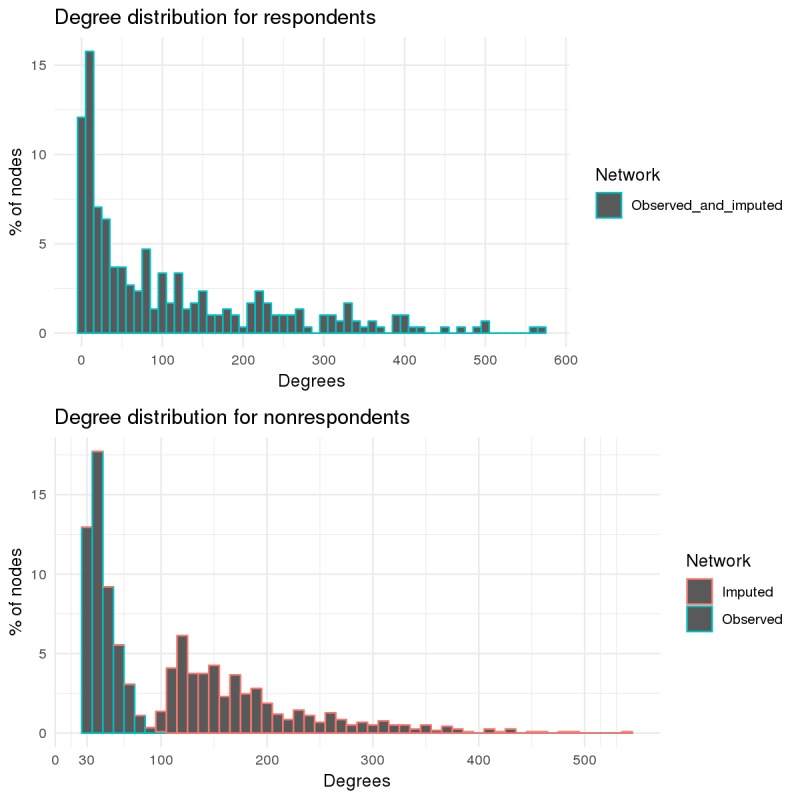
Degree distributions of respondents (top) and nonrespondents (bottom) in the observed and imputed networks. The respondent degree distributions in the top graph are identical because the imputation does not impact respondent ties. The degrees are binned together in sets of size 10.

The degree distributions for the respondents and nonrespondents in the observed network and 1 randomly selected imputed network are shown in Figure 4. The respondents have identical distributions in the observed and imputed graphs because the imputation does not impact respondent ties. We also observed that 11.7% (35/298) of the respondents had no friendships with anyone in the imputation sample, a consequence of excluding nonrespondents who did not meet our boundary specification.

The second consequence of our boundary specification is that we see that the minimum number of friendships for nonrespondents is 30. In addition, on the observed network, we noticed that numbers of friendships between nonrespondents had a much narrower range (30-100). After the imputation, however, we see that the degree distribution of nonrespondents is much more right-skewed, comparable with the degree distribution of the respondents. As we have no reason to believe that the respondents and nonrespondents should have different degree distributions, this correspondence in shape after imputation is a positive sign, indicating that our approach is reasonable in this sense. The left side of the imputed nonrespondent distribution does not resemble that of the respondents, but this is to be expected, given the degree-based boundary specification we imposed for selection of nonrespondents.

### Peer Change Agent Identification

#### Peer Change Agent Identification on the Observed Network

On the observed network, both the PCA sets of size 300 contained a mix of respondents and nonrespondents, with the exact breakdown varying by algorithm; the set for eigenvector centrality contained 62.0% (186/300) nonrespondents and for keyplayer contained 66.0% (197/300) nonrespondents. Recall that nonrespondents comprise 66.3% (587 of 885 nodes) of our sample. Thus, when the unobserved ties were treated as nonexistent, the proportion of nonrespondents in the PCA sets was higher than their proportion in the observed network for eigenvector centrality, and about the same for keyplayer. Although it might seem surprising that the proportion of nonrespondents who were selected as PCAs without the imputation was high, it makes sense in light of our degree-based boundary specification, which selected nonrespondents who had high connectivity with Chicago YBMSM, and were thus likely to be in critical positions. Had we not specified a boundary, nonrespondents would have comprised over 99.9% of the whole sample.

#### Peer Change Agent Identification on the Imputed Networks

**Figure 5:**
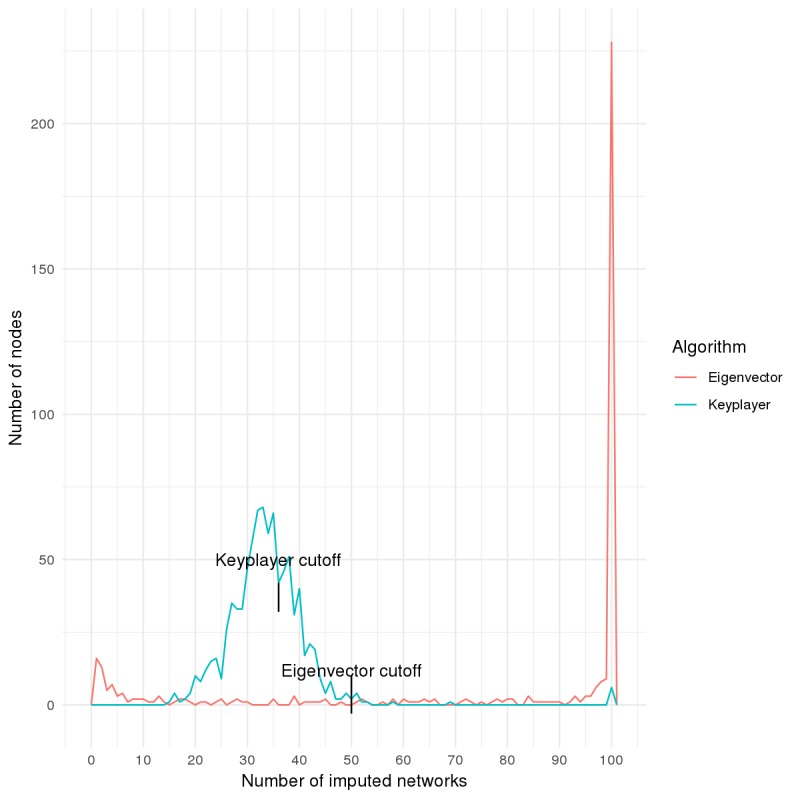
Distribution of the number of nodes selected as peer change agents on the imputed networks, conditional on their being selected at least once. This figure also illustrates the cutoff point for each algorithm, which is used to determine the sufficiency condition of peer change agent selection for each algorithm across the hundred imputations. KP: keyplayer; EV: eigenvector.

Across the 100 imputed networks, Figure 5 shows the distribution of PCAs identified by each algorithm. For eigenvector and keyplayer, 390 and 885 nodes, respectively, were selected at least once. Thus, we see a fundamental difference between the 2 measures in terms of their stability in node selection. Eigenvector centrality is a node-level algorithm, and it demonstrated a strong tendency to identify the same nodes as critical across all 100 imputations. In other words, the modal number of imputations for nodes that were selected at least once was 100. The keyplayer algorithm showed much less stability; the modal number of imputations for nodes that were selected at least once was 33. Summary statistics of the number of times PCAs were identified across all imputations are given in Table 3.

For subsequent analyses, we adopted a sufficiency condition, illustrated as the cutoff point in Figure 5. For each measure, this cutoff point was a value that yielded the smallest PCA set that was closest in size to our desired value of 300. We refer to PCA sets that meet the sufficiency condition for a given algorithm as sPCAs. With eigenvector centrality, 301 individuals—consisting of 22.9% (69/301) respondents and 77% (232/301) nonrespondents—were selected on at least 50 imputed networks. With keyplayer, 312 individuals—consisting of 35.2% (110/312) respondents and 64.7% (202/312) nonrespondents—were selected on at least 36 imputed networks. There were 115 sPCA individuals (20 respondents and 95 nonrespondents) that met the sufficiency criterion by both algorithms and 498 unique sPCA individuals selected by at least one algorithm (100 respondents and 398 nonrespondents).

We also found that nonrespondent sPCAs selected using eigenvector had a minimum of 44 friendships with respondents, whereas those selected using keyplayer were friends with a minimum of 30 respondents, which is the same as our boundary for nonrespondent inclusion. Thus, eigenvector only selected nonrespondent PCAs who were well above the boundary specification, whereas keyplayer did not. This suggests that eigenvector is less affected by the boundary specification for nonrespondent inclusion.

**Table 3:** Mean number of times that peer change agents were selected on the 100 imputed networks, conditional on their being selected at least once.

PCA^a^ identification algorithm	Number of times a node was identified as a PCA, mean (SD)	Number of times a respondent appeared as a PCA, mean (SD)	Number of times a nonrespondent appeared as a PCA, mean (SD)
Eigenvector centrality	76.9 (36.8)	99.3 (5.6)	72.1 (38.9)
Keyplayer	33.9 (8.3)	34.9 (11.7)	33.4 (5.9)

^a^PCA: peer change agent.

**Figure 6:**
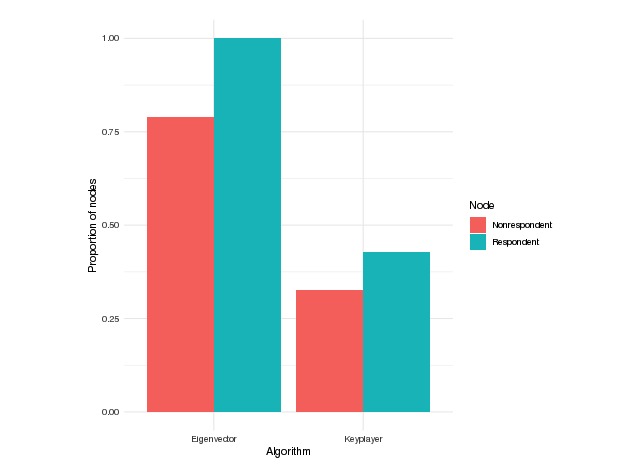
Proportion of nodes selected as sPCAs (peer change agents who met the sufficiency condition for each of the two peer change agent identification algorithms) on the imputed networks who were also selected as peer change agents on the observed network.

#### Comparison of Peer Change Agents Identified on the Observed and Imputed Networks

We found that all 69 respondents (100%) selected by eigenvector as sPCAs on the imputed networks were also selected as PCAs on the observed networks (Figure 6). Eigenvector selected 232 nonrespondent sPCAs on the imputed network, of whom 78.9% (183/232) appeared as PCAs on the observed network. Keyplayer selected 110 respondent sPCAs on the imputed networks, of whom 42.7% (47/110) appeared as PCAs on the observed network. Among nonrespondents, keyplayer selected 202 nonrespondents as sPCAs on the imputed networks, of whom 32.6% (66/202) appeared as PCAs on the observed network. We thus observe that eigenvector is much less sensitive to the imputation, as per our definition above, than keyplayer. Keyplayer’s higher sensitivity to the imputation might be because it is a set-based algorithm, and it attempts to select nodes that collectively span the breadth of the network, making the identification of a given node dependent not just on its local social environment but on the entirety of the network.

Although which of the 2 PCA-identification algorithms provides the true set of individuals in PCA positions (which is unknown) is not known, the contrasts between them among the various measures considered—stability, sensitivity, and effect of the boundary specification—stand out.

## Discussion

### Principal Findings

This paper presented a novel approach to select candidate PCAs on partially observed Facebook networks of YBMSM in Chicago, with the goal of developing a pipeline in the future that allows data from social networking sites to be used for peer health interventions. We discussed several challenges in operationalizing such an intervention, including methods to address the large amount of unobserved network data, and 2 PCA identification algorithms that are consistent with our goal of diffusing prevention information through individuals situated at critical positions in the network. We found that eigenvector centrality was far less sensitive to the imputation than keyplayer, consistent with a previous result [71]. We also found that relative to keyplayer, eigenvector had a relatively core set of stable PCAs across the imputed networks. Eigenvector centrality was also less affected by the relational boundary for nonrespondent inclusion, which was necessary given the large number of nonrespondents that were sampled. These findings lead us to conclude that eigenvector centrality might be better suited for identifying PCAs in our study. It is worth noting that we cannot know which algorithm produces the correct set of PCAs, but such a systematic evaluation of the properties of each algorithm, given that our data that were MNAR, can guide our intervention planning.

There are several underlying considerations behind this conclusion that merit discussion. Although peer-based interventions have shown promise in public health [72-75], their efficacy for HIV prevention has been limited in vulnerable populations [22,23]. The use of digital technologies to compile social network data and the application of formal social network analysis to identify PCAs may improve the efficacy of interventions [23,40]. Despite increased use of newer social network sites among younger people, Facebook use remains high in the general population [21], including YBMSM in Chicago. Recently collected data in a follow-up study have shown that although almost 100% of recruited YBMSM in Chicago used Facebook, fewer than 40% used Instagram and Snapchat and approximately 20% had profiles on Twitter, Jack’d, or Grindr. Thus, Facebook data enabled us to broadly characterize the social networks of YBMSM to identify their potential influencers. We, however, faced many unanticipated challenges in using Facebook data for identifying PCAs, and researchers using Facebook or alternate social media platforms may experience some of the same challenges we did. A schema to handle the limitations encountered here may benefit peer-based health research; our case study is a step forward in the development of such a schema.

### Limitations

One important limitation of the study is that no explicit homophily parameters were included in the imputation model. Recall that unobserved nonrespondent-nonrespondent friendships have to be imputed from the observed respondent-nonrespondent friendships. Many of the key individual attributes, including age, sex at birth, residence, and race (or ethnicity), either defined or were closely related to the inclusion criteria for respondents. As is described in the Results, nonrespondents who met the boundary specification were of comparable age to the respondents, almost all identified as male on their Facebook profiles, and a majority identified Chicago as their place of residence. Given the extent to which age, gender, and residence overlapped between the respondents and nonrespondents, it was not possible to meaningfully measure homophily with respect to these attributes. In addition, it was not possible to measure homophily on race/ethnicity as this attribute was not available for most nonrespondents. The imputation model also did not include any parameters that measured higher order network structure. Ideally, an ERGM fit to Facebook data would include higher order effects such as triad closure, as Facebook algorithms encourage individuals with common friends to become friends with each other. Our extensive efforts to incorporate terms for triad closure used existing parameters that were developed on sparser networks (eg, shared partner statistics), and they were not successful. (Information on the triad closure models we explored is provided in Multimedia Appendix 1.) More theoretical work may be needed to identify parameters that can model higher order effects in large networks with missing data. Our efforts, however, did reveal interesting new findings about the potential limits of using existing methods to model triad closure in large networks.

### Future Research Directions

Future research directions include modeling analyses to identify PCAs in a follow-up wave and assessing the extent to which PCA sets overlap between the 2 waves. This is important because training PCAs requires considerable upfront investment, and it has been observed in some networks that critically positioned individuals turn over within a year [76]. Therefore, identifying PCAs that persist over time might be more cost-effective, if they can be reliably found. In addition, many online environments besides Facebook are now used for social networking. As peer-based health interventions expand in scope, it may be valuable to consider alternative social media platforms as they may prove to be more effective with specific populations. Not all communication, however, occurs through such online media. Identification of friends and relational kin from offline data remains an important avenue for research. More theoretical work that explicitly accounts for online and *natural* social environments may improve our understanding of how to identify PCAs more accurately. Follow-up studies that address these considerations are in progress. Finally, Bayesian ERGMs to impute network unobserved data have been proposed [77] and might provide an alternate method to reconstruct the unobserved networks. (Details on related methodological approaches to impute missing network data, and why we selected the approach used here, are provided in Multimedia Appendix 1).

### Conclusions

This study is an interdisciplinary examination of a recruitment strategy of individuals located at critical positions in a large social network. Our overarching goal was to find a set of PCAs who maximize the possibility of success of our intervention while understanding the constraints that our data imposed. As Facebook and other online social media are increasingly used in creative ways to influence health behavior, our case study will help researchers anticipate some of the underlying difficulties as they plan their studies. If the challenges we described are unavoidable, our experiences could provide useful heuristics to maximize the potential for peer-based health interventions to succeed.

## Appendix

Multimedia Appendix 1 Detailed study background.

## Abbreviations

ERGM: Exponential Random Graph Model
MAR: missing at random
MCAR: missing completely at random
MCMC: Markov Chain Monte Carlo
MNAR: missing not at random
PCA: peer change agent
PrEP: pre-exposure prophylaxis
RDS: respondent-driven sampling
sPCA: peer change agents who met the sufficiency condition for each of the two peer change agent identification algorithms
YBMSM: young black men who have sex with men
