# Supplementary material for: Using Partially-Observed Facebook Networks to Develop a Peer-Based HIV Prevention Intervention: Case Study
Source: J Med Internet Res. 2018 Sep 14;20(9):e11652. doi: 10.2196/11652 (PMC6231846; doi:10.2196/11652)
Supplement: Multimedia Appendix 1 [file jmir_v20i9e11652_app1.pdf]

# Multimedia Appendix 1:
# Detailed Study Background

**TABLE OF CONTENTS**

**INTRODUCTION** **3**

**BACKGROUND** **3**
HIV among young Black men who have sex with men 3
Peer network interventions for HIV prevention 3
Identification of peer change agents (PCAs) 4
PCA identification measures of interest 4
Adapting Facebook data for prevention research 4
Partially observed network data: concepts and terminology 5
PCA identification on partially observed network data 5
Reconstructing unobserved networks 6
Reconstructing nonrespondent-nonrespondent data 6

**Imputation model: Convergence of Triad Closure Terms** **6**

**References to the Appendix** **7**

# Introduction

In this Appendix, we describe several details that are not in the main body of the paper. We present a more detailed background on peer-based health interventions, methodological principles important to the identification of candidate peer change agents (PCAs), and provide mathematical definitions of the two PCA identification algorithms we used. We discuss missing network data imputation techniques in more depth, including methods to identify PCAs when networks are partially observed. We also present details on our efforts to include higher order triad closure terms in our imputation model described in the main body of the paper.

# Background

## HIV among young Black men who have sex with men (YBMSM) in Chicago

Even as new HIV infections in the United States have stabilized over the past decade, YBMSM in the U.S. have continued to experience rising HIV incidence over this period [1]. In Chicago, the number of new HIV infections among YBMSM between 13- 29 years of age from 2004-2014 was annually at least five times higher than that for their White counterparts [2]. Reducing HIV incidence among YBMSM is an urgent public health priority.

Pre-exposure prophylaxis (PrEP) is a novel biomedical intervention that has been shown to substantially reduce acquisition of HIV infection in multiple high-risk populations [3]. Among MSM adherent to PrEP, an efficacy of over 90% has been estimated [3,4], and the US Centers for Disease Control and Prevention (CDC) have recommended its use [5]. Increasing the use of PrEP to reduce HIV incidence among YBMSM is an official strategy of the Chicago Department of Public Health. Despite recommendations for increasing PrEP uptake, use of PrEP among MSM in Chicago remains low [6,7]. Since individual-level interventions have had limited success in increasing PrEP use among YBMSM [8], we apply a social network approach here.

## Peer network interventions for HIV prevention

Network interventions, particularly those that are channeled through peers, have been found to be efficacious for HIV, both in the U.S. and globally [9]. An early example of prevention through diffusion of messaging was the “Stop AIDS” program in San Francisco during the early days of the HIV epidemic in the 1980s [10–12]. This program built upon small group communication and diffusion of information theories [13]. The diffusion was launched by training a small number of outreach workers, who conducted small group meetings in the gay neighborhoods of San Francisco. A well-respected HIV-positive individual in the community led these sessions, which were attended by gay and bisexual men. From 1985 to 1987, Stop AIDS reached over 30,000 men, and coincided with a marked decline in the number of new HIV infections [11]. A variant of this model was successfully applied to reduce sexual and drug-use behaviors related to HIV transmission [14], and was found to be effective among male prostitutes [15], in addition to diverse MSM populations in the United States [16–19], particularly Black MSM [20]. Thus, peer-based HIV interventions have shown promise for diverse high-risk populations. However, other studies have found conflicting results. For instance, the Community Popular Opinion Leader (C-POL) HIV intervention, where POLs were recruited and trained as behavior change endorsers [19], found that this peer-based intervention did not produce greater behavioral change than the control setting [21]. Some methods to improve the efficacy of this intervention have been proposed, including using digital methods to compile more accurate network data, and using formal network analyses to identify peer leaders [22].

## Identification of peer change agents (PCAs)

One challenge in the design of PCA interventions is the identification of individuals who would be effective disseminators of the intervention. While traditional PCA selection has used an ensemble of methods, including self-selection, peer nomination, and ethnographic observation [23], recent work has suggested that biobehavioral interventions are most likely to be effective when they account for the network structure of high risk individuals [24]. Such structural network assessments use formal mathematical and computational techniques, and position scores are computed for individuals, or ensembles of individuals [25]. It has also been argued that a PCA identification procedure is most successful when the type of flow process that is of interest is taken into account [26]. Following this argument, we apply two computational algorithms which are well-suited to situations where the underlying flow process involves diffusion of information: eigenvector centrality [27], and keyplayer positive [28]. In a recent study, we selected influential individuals by applying these two measures to the observed Facebook networks of YBMSM in the first two waves of our study. We found that individuals who were unaware of PrEP at baseline but became aware at follow-up had substantially more friendships with the influential nodes we identified than individuals who remained unaware of PrEP at both waves [7].

### PCA identification measures of interest: Eigenvector Centrality and Keyplayer Positive

The eigenvector centrality of a network is defined as the principal eigenvector of the adjacency matrix defining the network, as given by $\lambda\boldsymbol{v} = \boldsymbol{A}\boldsymbol{v}$, where $\boldsymbol{A}$ is the adjacency matrix of the network, $\lambda$ is the constant eigenvalue, and $\boldsymbol{v}$ is the eigenvector [27]. Each node $i$ receives a score that is equal to the $i$th component of the principal eigenvector. High scoring nodes are those that are connected to others that are themselves high scorers [26]. Eigenvector assumes that the flow process of interest moves through the network via unrestricted walks. It describes a mechanism where one node can impact all of its neighbors simultaneously [26], and has therefore been used in public health applications that utilize peer influence [29–31]. Eigenvector centrality (henceforth "eigenvector") is thus consistent with our application of PrEP-related information dissemination.

The keyplayer positive algorithm – henceforth referred to as "keyplayer" – is a set-based measure, reflecting the idea that the optimal set may not be necessarily composed of nodes who have the highest individual scores [28]. Keyplayer defines the cohesion between the members of a set $K$ in the nodes $V$ of a network, and the remainder of nodes in the network $V-K$, where cohesion is defined as $C_K = \sum_{j\in V-K} \bigcup_{i\in K} a_{ij,}$where $a_{ij}$ is 1 if nodes $i$ and $j$ are adjacent to each other and 0 otherwise), and $\bigcup a_{ij}$ is a maximum aggregation function equal to the maximum number of nodes in $V-K$ to which members of $K$are adjacent [28]. To find the optimal keyplayer set $K$, an optimization algorithm that starts with a randomly selected set $S$ is used, which computes $C_K$ by swapping each node $s \in$ S, with node $v \in V - S$ to compute a new cohesion score $C'_K$. The nodes $s$and $v$ are swapped only if $C'_K > C_K$, and the process is repeated until an optimal set is found [28]. The keyplayer set consists of individuals who are maximally connected to individuals in the network. Thus, passing information through the keyplayer set minimizes the social distance it has to travel to reach the maximum number of members of a social network. Keyplayer is thus an ideal choice for diffusing PrEP-related information, and it has been used in related public health applications [32,33].

## Adapting Facebook data for prevention research

Facebook communities are now being used to promote health behaviors such as obesity control [34], smoking cessation [35], and HIV/AIDS prevention [36]. To identify PCAs from a set of potential

influencers of Chicago YBMSM, we utilize Facebook data from "uConnect", a population-based longitudinal cohort study in Chicago that aims to study the role of social support networks of YBMSM on risk and risk-reduction practices to reduce new HIV infections [6]. Along with information on detailed biobehavioral characteristics assessed through an in-person survey, the Facebook friend lists of consenting uConnect participants were downloaded. We refer to individuals who appeared in our study as friends of one or more respondents, but who were not enrolled in the study themselves, as "nonrespondents." We are able to match nonrespondents who are named by more than one respondent, and because Facebook ties are undirected, we observe ties between nonrespondents and respondents. The Facebook friendships between pairs of nonrespondents, however, were unobserved. Our data on these Facebook networks are therefore incomplete, making it difficult to use these data to identify structurally positioned individuals most suitable for diffusion innovation that impacts HIV prevention.

There are additional complications in using Facebook data for prevention research. First, Facebook networks tend to be large, since individuals tend to have many Facebook friends. Second, although all network research must deal with issues in specifying the boundary that defines the nodes of interest, such issues might be pronounced when Facebook data are used, because Facebook networks combine individuals from many different components of one's life (compared to, say, professional collaborations in an organizational setting). Facebook friends are also not constrained by geography, or a high level of meaningful interaction. Thus, the ties can represent very heterogeneous relationships. Third, Facebook profiles do not consistently contain all of the attribute information that determine homophily or other social network effects. All of these factors compound the difficulties of using partially observed networks for a health intervention that would exist in other network studies using datasets other than Facebook.

## Partially observed network data: theoretical concepts and terminology

It is imperative to examine how the unobserved network can be reconstructed, given that we are using partially observed Facebook networks. Therefore, we must first define the nature of "missingness" in a partially observed dataset. Following the widely used convention developed by Rubin [37], data are called "missing completely at random" (MCAR) when the missingness depends neither on the observed data nor the unobserved data. Data are "missing at random" (MAR) if the probability of missingness does depend on the observed data but not the unobserved data [38,39], and "missing not at random" (MNAR) if the probability of missingness depends on the unobserved data [38]. Huisman (2009) provides an interpretation of these definitions for networks with unobserved edges [40], such as ours. In particular, if the probability of missingness is related to the existence of the edges, the data are MNAR [40].

## PCA identification on partially observed network data

There is a growing body of literature on identifying critical nodes on networks that are incompletely observed [41–47]. This literature can be divided into two parts: 1) papers that examine scenarios with false positive ("spurious") and false negative ("missing") nodes [43,48–50], and, 2) those that consider spurious and missing edges [46,47]. The findings suggest a "smooth and consistent decline" in the accuracy of PCA identification as the number of missing nodes increases [51]. The conclusions with missing edge sets are mixed. On one hand, Borgatti et al. use Erdős–Rényi graphs to find that missing edges have a sizeable negative effect on the accuracy of identified key nodes, which increases in magnitude as the number of missing edges increases, and contend that missing edges are more damaging than the spurious [46], though Wang et al. [47] use two empirical networks to suggest that this result may not be generally true.

While these studies are instructive, they are not perfectly comparable to our case. Borgatti and Carley et al. considered a random Erdős–Rényi network with randomly missing nodes and edges [45,46], a pattern that fits the MCAR definition above. Wang et al. consider empirical networks, but with only 5 percent of

nodes and edges that are MCAR. Smith and Moody considered the impact of random [51] and non-random [52] node removal on network statistics in a number of directed and undirected empirical networks, but not that of missing edges. Kossinets considers bipartite networks with a number of mechanisms for missing edge configurations in a bipartite scientific collaboration network, but the network statistics he considered are not applicable to our PCA identification measures [53]. Our data describe an empirical network with a large amount of missing data and an MNAR structure of missingness; more details are in Section 4 below.

### Reconstructing unobserved networks

The preferred approach for recovery of missing data depends on the type of missingness. One early technique for working with missing network data is the method of "reconstruction", proposed by Stork and Richards [54], and examined in more recent work [40,55]. This method allows for unobserved ties from nonrespondents to respondents to be reconstructed, based on the report provided by the respondent on this tie. However, as the authors themselves argue, this technique cannot be used directly to impute unobserved ties between nonrespondents.

Huisman (2009) presented a sparse network of adolescent friendships with multiple mechanisms of missing nodes and edges and at varied proportions of missingness [40]. He then imputed the missing data using four different techniques based on sampling from unconditional distributions, and measured the effect of this imputation on some common statistics of network structure, but not the type of node influence processes that we are interested in.

Our interest is in the reliable identification of PCAs, given an incompletely observed network, where the data are MNAR. A method to impute unobserved edge data using conditional distributions was proposed by Robins [44]. This method utilized exponential random graph models (ERGMs) and considered two cases: one where the goal was to model the full network structure but nonrespondents were MAR, and a second where the principal focus was to understand the respondent-only structure, albeit while using the observed information regarding ties to nonrespondents. The networks discussed by Robins et al. were directed, while the Facebook networks that are of interest to us are undirected. Moreover, Robins et al. applied the imputation method to impute unobserved data between respondents and nonrespondents, not between pairs of nonrespondents, as is needed to characterize the YBMSM network of interest.

### Reconstructing nonrespondent-nonrespondent data

To impute the unobserved friendships, we use a method developed by Handcock and Gile [56], where imputation is based on multiple imputations of the full network conditional upon the observed network. In contrast, Huisman's method used unconditional distributions [40]. Thus, we apply a likelihood-based imputation technique where all the observed and unobserved data are modeled simultaneously [44]. We then apply two different algorithms (eigenvector and keyplayer) to identify PCA sets on these imputed networks. We compare PCAs selected by these algorithms in the main body of the manuscript.

## Imputation model: Convergence of Triad Closure Terms

Triad closure terms have been found to be important in predicting missing network data [57]. Our extensive efforts to incorporate terms for triad closure, however, all failed to converge. These began with a parameter for the count of triangles in the network, consistent with previous work [58,59]; this model was found to be "degenerate," as defined in earlier work [59,60]. We then explored a variety of models including geometrically-weighted edgewise shared partner (GWESP) terms, alone and in combination with geometrically-weighted dyadwise shared partner (GWDSP) terms [61]. These consisted of models in

which decay parameters were fixed and those in which they were estimated. All models were degenerate. We next attempted to constrain the model from diverging from observed network density by including a variety of additional *degree* terms, which specify the expected number of nodes with a specific degree, in conjunction with GWESP and/or GWDSP terms, with no improvement in model convergence. We take two lessons from this exercise. First, the terms commonly used within ERGMs for capturing triadic effects may not be as appropriate for high-degree networks like Facebook as they are for the lower-degree networks to which they have traditionally been applied. Second, that the presence of missing data may make these terms especially challenging to estimate for networks of the size explored here. Thus, we found something new about the potential limits of existing parameters in modeling large, dense networks. Both areas identified above are where future methodological research is needed.

## References to the Appendix


1. Prejean J, Song R, Hernandez A, Ziebell R, Green T, Walker F, et al. Estimated HIV incidence in the United States, 2006-2009. PLoS One. 2011;6. doi:10.1371/journal.pone.0017502
2. Benbow N. HIV Trends and Characteristics in Chicago. Chicago: Third Coast Center for AIDS Research Workshop with Chicago Department of Public Health. Surveillance Data: A Tool for HIV Researchers; 2016.
3. Grant RM, Lama JR, Anderson PL, McMahan V, Liu AY, Vargas L, et al. Preexposure chemoprophylaxis for HIV prevention in men who have sex with men. N Engl J Med. 2010;363: 2587–2599. doi:10.1056/NEJMoa1011205
4. Anderson PL, Glidden D V., Liu A, Buchbinder S, Lama JR, Guanira J V., et al. Emtricitabine-Tenofovir Concentrations and Pre-Exposure Prophylaxis Efficacy in Men Who Have Sex with Men. Science Translational Medicine. 2012. p. 151ra125-151ra125. doi:10.1126/scitranslmed.3004006
5. Centers for Disease Control and Prevention. Prexposure prophylaxis for the prevention of HIV infection in the United States. In: A Clinical Practice Guideline [Internet]. 2014. Available: http://www.cdc.gov/hiv/pdf/prepguidelines2014.pdf
6. Khanna AS, Michaels S, Skaathun B, Morgan E, Green K, Young L, et al. Preexposure Prophylaxis Awareness and Use in a Population-Based Sample of Young Black Men Who Have Sex With Men. JAMA Intern Med. 2016;176: 136–8. doi:10.1001/jamainternmed.2015.6536
7. Khanna AS, Schumm P, Schneider JA. Facebook network structure and awareness of preexposure prophylaxis among young men who have sex with men. Ann Epidemiol. 2017;27: 176–180. doi:10.1016/j.annepidem.2016.11.006
8. Hosek SG, Siberry G, Bell M, Lally M, Kapogiannis B, Green K, et al. The acceptability and feasibility of an HIV preexposure prophylaxis (PrEP) trial with young men who have sex with men. J Acquir Immune Defic Syndr. 2013;62: 447–56. doi:10.1097/QAI.0b013e3182801081
9. Simoni JM, Nelson KM, Franks JC, Yard SS, Lehavot K. Are peer interventions for HIV efficacious? A systematic review. AIDS Behav. 2011;15: 1589–95. doi:10.1007/s10461-011-9963-5
10. Wohlfeiler D. Community organizing and community building among gay and bisexual men: The STOP AIDS Project. In: Minkler M, editor. Community Organizing and Community Building for Health. New Brunswick, NJ: Rutgers University Press; 1998. pp. 230–243.
11. Bertrand JT. Diffusion of Innovations and HIV/AIDS. J Health Commun. 2004;9: 113–121. doi:10.1080/10810730490271575
12. Rogers EM. A Prospective and Retrospective Look at the Diffusion Model. J Health Commun. 2004;9: 13–19. doi:10.1080/10810730490271449
13. Singhal A, Rogers EM. Combating AIDS : communication strategies in action [Internet]. Sage Pub; 2003. Available: https://catalog.lib.uchicago.edu/vufind/Record/4911764

14. Latkin CA, Sherman S, Knowlton A. HIV prevention among drug users: outcome of a network-oriented peer outreach intervention. Health Psychol. 2003;22: 332–9. Available: http://www.ncbi.nlm.nih.gov/pubmed/12940388
15. Miller RL, Klotz D, Eckholdt HM. HIV prevention with male prostitutes and patrons of hustler bars: replication of an HIV preventive intervention. Am J Community Psychol. 1998;26: 97–131. Available: http://www.ncbi.nlm.nih.gov/pubmed/9574500
16. Kelly JA, St Lawrence JS, Diaz YE, Stevenson LY, Hauth AC, Brasfield TL, et al. HIV risk behavior reduction following intervention with key opinion leaders of population: an experimental analysis. Am J Public Health. 1991;81: 168–71. Available: http://www.ncbi.nlm.nih.gov/pubmed/1990853
17. Kelly JA, St Lawrence JS, Stevenson LY, Hauth AC, Kalichman SC, Diaz YE, et al. Community AIDS/HIV risk reduction: the effects of endorsements by popular people in three cities. Am J Public Health. 1992;82: 1483–9. Available: http://www.ncbi.nlm.nih.gov/pubmed/1443297
18. Kelly JA, Murphy DA, Sikkema KJ, McAuliffe TL, Roffman RA, Solomon LJ, et al. Randomised, controlled, community-level HIV-prevention intervention for sexual-risk behaviour among homosexual men in US cities. Community HIV Prevention Research Collaborative. Lancet (London, England). 1997;350: 1500–5. Available: http://www.ncbi.nlm.nih.gov/pubmed/9388397
19. Kelly JA. Popular opinion leaders and HIV prevention peer education: resolving discrepant findings, and implications for the development of effective community programmes. AIDS Care. 2004;16: 139–50. doi:10.1080/09540120410001640986
20. Hosek SG, Lemos D, Hotton AL, Fernandez MI, Telander K, Footer D, et al. An HIV intervention tailored for black young men who have sex with men in the House Ball Community. AIDS Care. 2015;27: 355–62. doi:10.1080/09540121.2014.963016
21. NIMH Collaborative HIV/STD Prevention Trial Group. Results of the NIMH collaborative HIV/sexually transmitted disease prevention trial of a community popular opinion leader intervention. J Acquir Immune Defic Syndr. 2010;54: 204–14. doi:10.1097/QAI.0b013e3181d61def
22. Schneider JA, Laumann EO. Alternative Explanations for Negative Findings in the Community Popular Opinion Leader Multisite Trial and Recommendations for Improvements of Health Interventions Through Social Network Analysis. JAIDS J Acquir Immune Defic Syndr. 2011;56: e119–e120. doi:10.1097/QAI.0b013e318207a34c
23. Valente TW, Pumpuang P. Identifying opinion leaders to promote behavior change. Health Educ Behav. 2007;34: 881–96. doi:10.1177/1090198106297855
24. Schneider JA, McFadden RB, Laumann EO, Prem Kumar S, Gandham SR, Oruganti G. Candidate change agent identification among men at risk for HIV infection. Soc Sci Med. 2012;75: 1192–1201. doi:10.1016/j.socscimed.2012.05.022
25. Schneider JA, Zhou AN, Laumann EO. A new HIV prevention network approach: sociometric peer change agent selection. Social Science and Medicine. Jan 2014: 192–202. doi:10.1016/j.socscimed.2013.12.034
26. Borgatti SP. Centrality and network flow. Soc Networks. 2005;27: 55–71. doi:10.1016/j.socnet.2004.11.008
27. Bonacich P. Power and Centrality: A Family of Measures. American Journal of Sociology. 1987. p. 1170. doi:10.1086/228631
28. Borgatti SP. Identifying sets of key players in a social network. Comput Math Organ Theory. 2006;12: 21–34.
29. Johnson K, Quanbeck A, Maus A, Gustafson DH, Dearing JW. Influence networks among substance abuse treatment clinics: implications for the dissemination of innovations. Transl Behav Med. 2015;5: 260–8. doi:10.1007/s13142-015-0327-y
30. Geissler KH, Lubin B, Marzilli Ericson KM. Access is Not Enough: Characteristics of Physicians Who Treat Medicaid Patients. Med Care. 2016;54: 350–358. doi:10.1097/MLR.0000000000000488

31. Gyarmathy VA, Caplinskiene I, Caplinskas S, Latkin CA. Social network structure and HIV infection among injecting drug users in Lithuania: gatekeepers as bridges of infection. AIDS Behav. NIH Public Access; 2014;18: 505–10. doi:10.1007/s10461-014-0702-6
32. Young AM, Jonas AB, Mullins UL, Halgin DS, Havens JR. Network structure and the risk for HIV transmission among rural drug users. AIDS Behav. NIH Public Access; 2013;17: 2341–51. doi:10.1007/s10461-012-0371-2
33. Cobb NK, Graham AL, Abrams DB. Social network structure of a large online community for smoking cessation. Am J Public Health. American Public Health Association; 2010;100: 1282–9. doi:10.2105/AJPH.2009.165449
34. Herring SJ, Cruice JF, Bennett GG, Rose MZ, Davey A, Foster GD. Preventing excessive gestational weight gain among African American women: A randomized clinical trial. Obesity (Silver Spring). 2016;24: 30–6. doi:10.1002/oby.21240
35. Thrul J, Klein AB, Ramo DE. Smoking Cessation Intervention on Facebook: Which Content Generates the Best Engagement? J Med Internet Res. 2015;17: e244. doi:10.2196/jmir.4575
36. Phillips G, Ybarra ML, Prescott TL, Parsons JT, Mustanski B. Low Rates of Human Immunodeficiency Virus Testing Among Adolescent Gay, Bisexual, and Queer Men. J Adolesc Health. 2015;57: 407–12. doi:10.1016/j.jadohealth.2015.06.014
37. Rubin DB. Inference and Missing Data. Biometrika. 1976;63: 581. doi:10.2307/2335739
38. Schafer JL, Graham JW. Missing data: our view of the state of the art. Psychol Methods. 2002;7: 147–77. Available: http://www.ncbi.nlm.nih.gov/pubmed/12090408
39. McKnight PE. Missing data : a gentle introduction [Internet]. Guilford Press; 2007. Available: https://catalog.lib.uchicago.edu/vufind/Record/6435017
40. Huisman M. Imputation of missing network data: some simple procedures. J Soc Struct. 2009;10.
41. Holland, PW; Leinhardt S. Structural implications of measurement error in sociometry. J Math Sociol. 1973;3: 85–111.
42. Burt RS. A note on missing network data in the general social survey. Soc Networks. 1987;9: 63–73. doi:10.1016/0378-8733(87)90018-9
43. Costenbader E, Valente TW. The stability of centrality measures when networks are sampled. Soc Networks. 2003;25: 283–307. doi:10.1016/S0378-8733(03)00012-1
44. Robins G, Pattison P, Woolcock J. Missing data in networks: Exponential random graph (p*) models for networks with non-respondents. Soc Networks. 2004;26: 257–283.
45. Carley KM, Reminga J, Borgatti S. Destabilizing dynamic networks under conditions of uncertainty. IEMC ’03 Proceedings Managing Technologically Driven Organizations: The Human Side of Innovation and Change (IEEE Cat No03CH37502). IEEE; 2003. pp. 121–126. doi:10.1109/KIMAS.2003.1245033
46. Borgatti SP, Carley KM, Krackhardt D. On the robustness of centrality measures under conditions of imperfect data. Soc Networks. 2006;28: 124–136. doi:10.1016/j.socnet.2005.05.001
47. Wang DJ, Shi X, McFarland DA, Leskovec J. Measurement error in network data: A re-classification. Soc Networks. 2012;34: 396–409. doi:10.1016/j.socnet.2012.01.003
48. Galaskiewicz J. Estimating point centrality using different network sampling techniques. Soc Networks. 1991;13: 347–386. doi:10.1016/0378-8733(91)90002-B
49. Carnegie NB, Goodreau SM, Liu A, Vittinghoff E, Sanchez J, Lama JR, et al. Targeting pre-exposure prophylaxis among men who have sex with men in the United States and peru: partnership types, contact rates, and sexual role. J Acquir Immune Defic Syndr. 2015;69: 119–125. doi:10.1097/QAI.0000000000000555
50. Johnson JC, Boster JS, Holbert D. Estimating relational attributes from snowball samples through simulation. Soc Networks. 1989;11: 135–158. doi:10.1016/0378-8733(89)90009-9
51. Smith JA, Moody J. Structural effects of network sampling coverage I: Nodes missing at random. Soc Networks. 2013;35: 652–668. doi:10.1016/j.socnet.2013.09.003
52. Smith JA, Moody J, Morgan JH. Network sampling coverage II: The effect of non-random missing data on network measurement. Soc Networks. 2017;48: 78–99.

doi:10.1016/j.socnet.2016.04.005
53. Kossinets G. Effects of missing data in social networks. Soc Networks. 2006;28: 247–268. doi:10.1016/j.socnet.2005.07.002
54. Stork D, Richards WD. Nonrespondents in Communication Network Studies: Problems and Possibilities. Gr Organ Manag. 1992;17: 193–209. doi:10.1177/1059601192172006
55. Huisman M, Steglich C. Treatment of non-response in longitudinal network studies. Soc Networks. 2008;30: 297–308. doi:10.1016/j.socnet.2008.04.004
56. Handcock MS, Gile KJ. Modeling social networks from sampled data. The Annals of Applied Statistics. 2010. pp. 5–25. doi:10.1214/08-AOAS221
57. Clauset A, Moore C, Newman MEJ. Hierarchical structure and the prediction of missing links in networks. Nature. 2008;453: 98–101. doi:10.1038/nature06830
58. Goodreau SM, Kitts JA, Morris M. BIRDS OF A FEATHER, OR FRIEND OF A FRIEND? USING EXPONENTIAL RANDOM GRAPH MODELS TO INVESTIGATE ADOLESCENT SOCIAL NETWORKS. Demography. 2009;46: p103-125. Available: http://offcampus.lib.washington.edu/login?url=http://search.ebscohost.com/login.aspx?direct=true&db=a9h&AN=36168694&site=ehost-live
59. Handcock MS. Assessing Degeneracy in Statistical Models of Social Networks [Internet]. 2003. Available: www.stat.washington.edu/handcock.
60. Chatterjee S, Diaconis P. Estimating and understanding exponential random graph models. 2011; doi:10.1214/13-AOS1155
61. Morris M, Handcock MS, Hunter DR. Specification of Exponential-Family Random Graph Models: Terms and Computational Aspects. J Stat Softw. 2007;24: 1–24. Available: http://www.jstatsoft.org/v24/i04
